# Preparation of ZnO Nanoparticles with High Dispersibility Based on Oriented Attachment (OA) Process

**DOI:** 10.1186/s11671-019-3038-3

**Published:** 2019-06-20

**Authors:** Dingding Cao, Sheng Gong, Xugang Shu, Dandan Zhu, Shengli Liang

**Affiliations:** School of Chemistry and Chemical Engineering, Zhongkai University of Agriculture and Engineering, Guangzhou, 510220 China

**Keywords:** Zinc oxide, Nanocrystals synthesis, Growth mechanism, Oriented attachment (OA), Suspension property

## Abstract

Understanding nanoparticle growth mechanisms is crucial for the synthesis of nanocrystals with desired biological and chemical properties. Growth of nanocrystals by oriented attachment (OA) is frequently reported as a method supplementary to the classical growth by Ostwald ripening (OR) process. In this work, ZnO nanoparticles (NPs) were prepared by wet chemical method. Size/shape evolution of ZnO NPs in ethanol solution was systematically studied using transmission electron microscopy (TEM), dynamic light scattering (DLS), and X-ray diffraction (XRD). In addition, a detailed process of the nanoparticle growth-based OA mechanism is discussed. Results revealed that reaction conditions affect size/shape of NPs and change their surface structure: prior to OA, the surface of adjacent particles transformed into their “rough” states. We proved that stability of the solution was significantly improved in this state. Such a state is important to design nanoparticles with high stability and as nano-suspensions with special physical and/or chemical properties. This state is a critical step in enhancing OA process.

**Electronic supplementary material:**

The online version of this article (10.1186/s11671-019-3038-3) contains supplementary material, which is available to authorized users.

## Introduction

ZnO nanoparticles (NPs) attract a lot of attention for fundamental studies and potential applications in different research areas: from physical chemistry to biomedical sciences [1]. ZnO NPs represent a versatile functional material, and their superior properties find current and potential applications in catalysts, transducers, semiconductors, microelectronics, textile, cosmetics, water treatment [2], etc. Additionally, ZnO NPs exhibit antimicrobial activity and anti-inflammation properties [3], providing more efficient, less expensive, and less toxic [4] alternatives to antibiotics and bactericides.

Variety of the synthesis routes for ZnO NPs is remarkable [5–7]. However, it is still challenging to control their crystalline structure, stability, and dispersibility in common solutions such as water and ethanol [8, 9]. As complexity of synthetic reactions increases, a thorough understanding of nanoparticle formation mechanism is needed [10, 11]. General mechanism is more or less understood. However, major gaps in understanding of oriented attachment (OA) as well as in the understanding of how particle structure changes still remain [12]. A lot of experimental data interpretation and description during OA crystallization are reported [13]. However, efforts to explain this phenomenon quantitatively and from a point of view of its mechanism started appearing in the literature only recently. Especially, understanding how NP performance in a suspension is affected by the particle morphology is lacking [12]. Control of ZnO NP stability, solubility, surface structure, shape, and aggregation properties represents some of the key roles for ZnO NP industrial and other practical applications [5]. As nano-industry develops, long-standing and traditional interpretations of particle formation mechanisms must be revisited.

This work focuses on the synthesis of highly stable suspension of ZnO nanoparticles (NPs) optimized by changing pH, reaction time, and growth temperature. The growth process of NPs (individual as well as their clusters) was monitored by high-resolution transmission electron microscopy (HR-TEM) and X-ray powder diffraction (XRD). This is the first study to report the effect of the reaction conditions on suspension and dispersion of ZnO NPs.

Relationship between particle structure and growth kinetics was determined by studying the OA process of crystal growth. This study provides a better understanding of nanoparticles growth from a physical-chemical point of view of stability, dispersibility, and suspension morphologies. ZnO NPs obtained in this work demonstrated excellent stability in suspensions, which can be widely used for practical applications.

## Methods

Zinc acetate dihydrate (Zn(O_2_CCH_3_)_2_(H_2_O)_2_) and sodium hydroxide (NaOH) were purchased from Shanghai Aladdin Biochemical Technology Co. (China). Absolute ethanol was obtained from Tianjin Damao Chemical Reagents Co. (China). All reagents were analytically pure and used as received without any further purification.

First, products were prepared at the following standard conditions: 60 °C synthesis temperature, 2 h duration, 7.22 and 3.73 mmol of NaOH and zinc acetate dihydrate, respectively, as initial starting material quantities. To study this reaction and to obtain the best product, the synthesis procedure was modified by changing precursor concentrations, reaction time, and temperature as well as pH. Final products were white precipitates (see Additional file 5: Table S1).

As it was discussed elsewhere [13–15], synthesis mixture was prepared from two different solutions: solution A and solution B; solution A contained 3.73 mmol of zinc acetate dihydrate dissolved in 40 ml of ethanol; solution B contained 7.22 mmol of NaOH dissolved in 320 μL of bi-distilled water and then in 25 mL of ethanol. Solution B was added dropwise to solution A under vigorous and constant stirring for 2.25 h at 45, 50, 55, 60, and 65 °C, after which solution was allowed to cool down to room temperature. As-synthesized ZnO samples were collected by centrifugation and washed thoroughly with pure ethanol. This procedure was repeated several times: ZnO NPs were re-dispersed in ethanol or dried at 60 °C for 2 h. All ZnO NPs were stored at room temperature. These samples were marked as samples 1–6, respectively. During the formation of NPs, the following reactions occurred [16]: (Zn(O_2_CCH_3_)_2_(H_2_O)_2_) reacted with NaOH in ethanol. Dehydrating properties of ethanol prevented the formation of zinc hydroxide [17].

Aging experiments were performed using experimental conditions of sample 4. Durations of aging experiments were 1, 1.5, 2.25, 6, 12, and 24 h. Samples were marked as samples 19–24, respectively. Another series of experiments were performed with different precursor concentrations: 1, 4, 7, 10, 14, and 18 mmol of Zn(O_2_CCH_3_)_2_(H_2_O)_2_) and 3.73, 5.22, 6.34, 7.46, 8.58, and 9.33 mmol of NaOH. These samples were marked as samples 7–18, respectively.

**Scheme 1:**
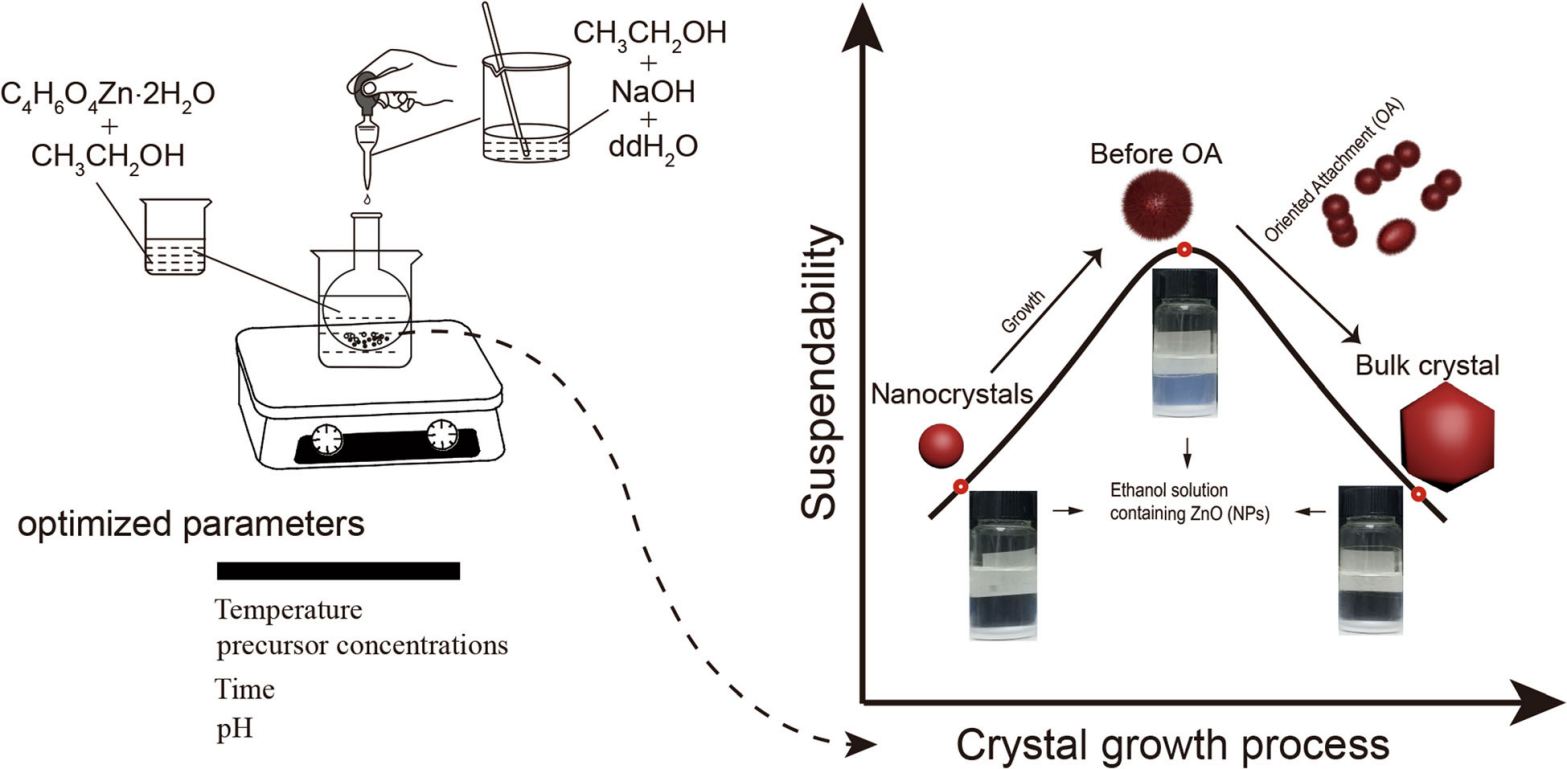
Schematic illustration of ZnO NPs synthesis as well as the suspendability study

A certain amount of the ZnO NPs was taken after washing and centrifugation and re-dispersed into a glass bottle (containing fresh ethanol) by ultrasonication and vigorous shaking. After that, dispersion and stability of samples were characterized visually during aging experiments, which lasted for 1, 7, 14, and 21 days. To determine the suspendability of the samples, the supernatant was subjected to the light absorbance measurements performed at *λ* = 370 nm [7, 18]. The synthetic process of ZnO NPs as well as the suspendability study is described in Scheme 1.

UV Lambda 370 ultraviolet-visible spectrometer from Perkin Elmer (Waltham, MA, USA) was used to measure sample absorbance at room temperature. Sample morphologies were characterized using scanning electron microscope (SEM, Hitachi, Tokyo, Japan) and FEI Tecnai G2 F20 high-resolution transmission election microscope (HR-TEM) coupled with energy dispersive X-ray spectroscopy (EDX) and selected area electron diffraction (SAED) (Thermo Fisher Scientific, Waltham, MA, USA). Crystal structures were detected using Smartlab X-ray powder diffractometer (XRD) with Cu Kαradiation (*λ* = 1.5418 Å) in the 2*θ* = 20–80°range with 5°/min scanning speed. Particle sizes of NPs in ethanol were obtained using dynamic light scattering (DLS) particle size analyzer (ELSZ-2, Otsuka Electronics Co., Osaka, Japan). ZnO NP suspensions were carefully sonicated prior to each experiment to minimize the aggregation effect.

## Results and Discussion

### Suspendability Analysis

**Fig. 1:**
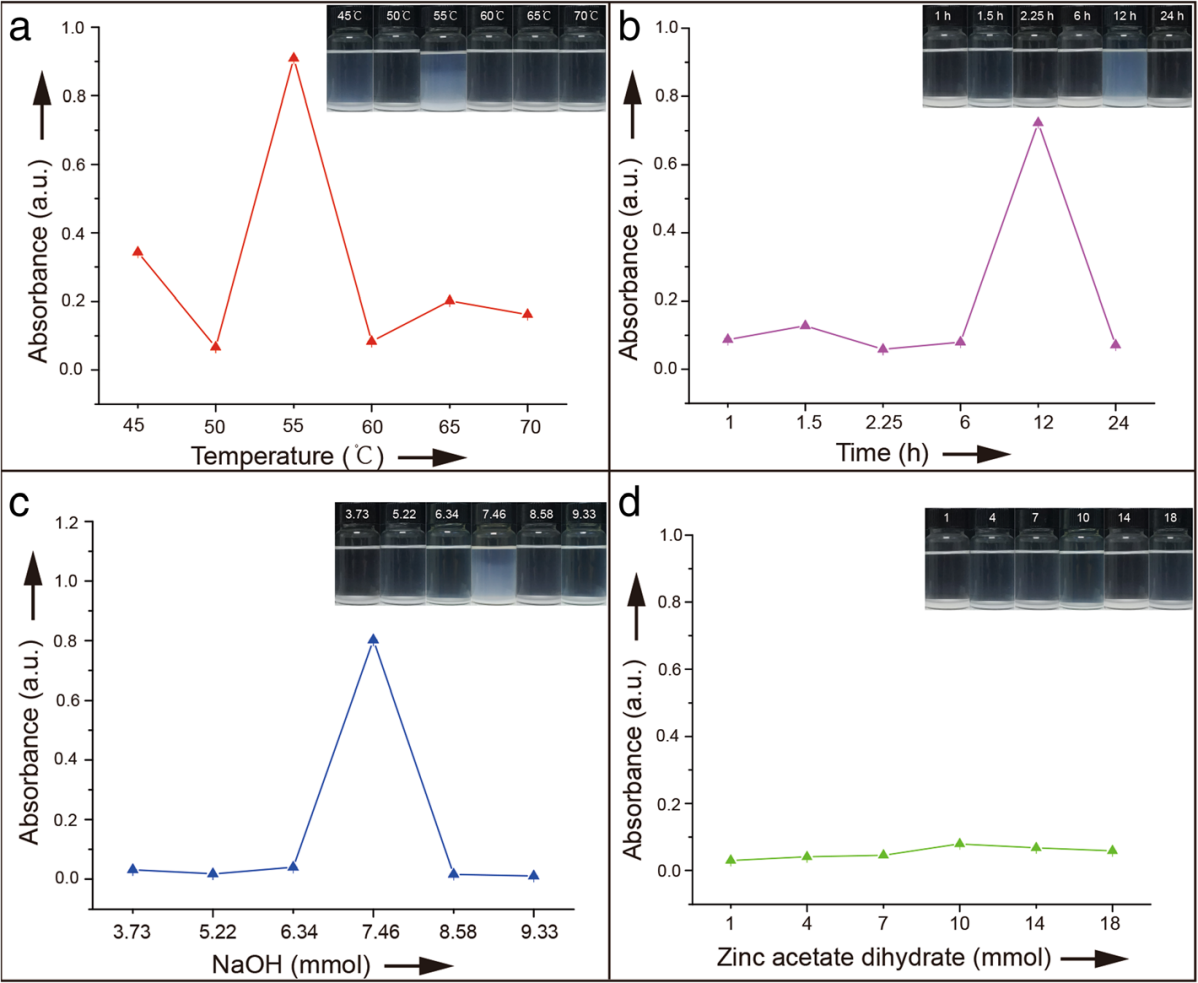
Absorbance of ZnO NPs in ethanol solution at *λ* = 370 nm obtained at different **a** reaction temperature, **b** reaction time, **c** moles of sodium hydroxide, and **d** moles of zinc acetate dihydrate, after 3 weeks of aging at room temperature

In practice, the decrease in turbidity is interpreted as a decrease in suspendability [19]. Here, the suspendability of NPs in ethanol solution was studied by turbidimetry [18]. Turbidity of different samples is clearly distinguishable by visual turbidity (see Fig. 1). In order to better digitize the sample turbidity difference, spectrophotometric techniques are commonly used [20, 21]. Generally, turbidity is measured using a wavelength that will not be absorbed by the suspended nanoparticles [18]. As shown in Additional file 1: Figure S1, the samples do not absorb at 370 nm. The obtained results measured at 370 nm are in good agreement with those observed by visual turbidity, i.e., at 370 nm, absorbance can reflect suspendability of the solution (Fig. 1). Furthermore, samples 3 and 21 were selected for zeta potential analysis, indicating that those with high turbidity have higher Z-potential (see Additional file 4: Figure S4).

Reaction time, temperature, and pH played a critical role in suspension properties. Desired suspension properties can be achieved only under certain reaction conditions, therefore, reaction factors and their combinations need to be optimized. Solutions obtained under different reaction conditions exhibited excellent stability as well as outstanding suspension performance (see Fig. 1a–c). When reaction conditions were 55 °C, 12 h, and 7.46 mmol of initial NaOH, particles exhibited excellent long-term suspension performance in ethanol. Further increase of the reaction time, temperature, and pH value resulted in particle precipitation and deterioration of suspension performance.

Contrary to the previously reported results [22], suspendability of ZnO NPs in this work was not affected by the precursor concentrations (see Fig. 1d). This result also contradicts to the classical crystal theory since the probability of particle collisions would be enhanced at higher concentrations. Results from this work proved that increased precursor concentration during non-classical crystallization is not a prerequisite for particles agglomeration.

Suspension properties of ZnO NPs in ethanol demonstrated an inverted U-shape curve as function of certain conditions. At longer reaction time, higher temperature and higher pH values, ZnO NP ethanol suspensions remained highly transparent. These variations further demonstrate changes of ZnO NPs surface structure. In general, surface characteristics of NPs strongly affect suspension appearance and properties of materials. They can lead to unique suspension morphologies (see Fig. 1) and long-term suspension performance. Our experiments proved that these colloids remained in a dispersed state for weeks. Thus, studying suspension morphology can provide useful information on OA processes and surface structure of NPs.

### XRD Analysis

**Fig. 2:**
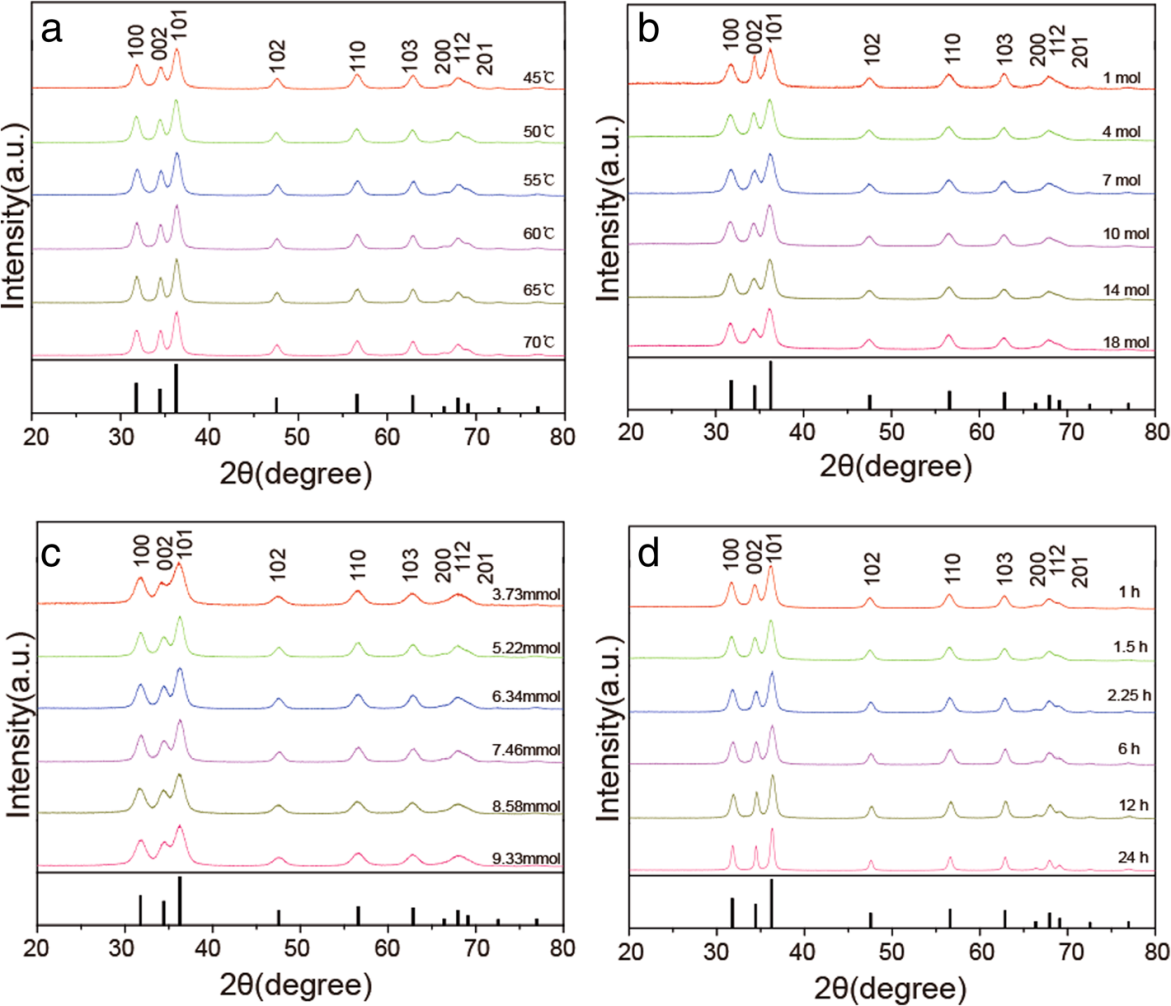
XRD patterns of ZnO NPs obtained at different **a** reaction temperature, **b** moles of zinc acetate dihydrate, **c** moles of sodium hydroxide, and **d** reaction time. XRD pattern of bulk ZnO (according to JCPDS no. 36-1451) is shown at the bottom of each set of XRD patterns

Diffraction peaks of all samples corresponded to hexagonal ZnO with wurtzite structure judging by the JCPDS card no. 36-1451 (see Fig. 2 and Additional file 2: Figure S2). No other phases, e.g., sphalerite, were observed. c-lattice constant calculated from the XRD peaks of sample 4 was 0.26 nm. All patterns had broadened reflections due to the small particle sizes.

Comparison of XRD patterns of samples obtained with different reaction times (shown in Fig. 2d) demonstrated that intensity of the (002) peak was enhanced for samples 11 and 12 indicating that nanorods grew along the *c*-axis. Changes in all other conditions did not affect XRD peak intensity. Nanoparticles demonstrated clear crystalline facets (see Fig. 5b). Average nanoparticles sizes analyzed using Scherrer equation [23] are shown in Fig. 2. Particle sizes of samples 1–21 and samples 22–24 were 5–15 nm and 10–100 nm, respectively. These values are consistent with TEM results shown in Fig. 5. These results also confirm that particle sizes were not the major factors causing different properties of the solution suspensions shown on Fig. 1.

### Morphological Analyses

**Fig. 3:**
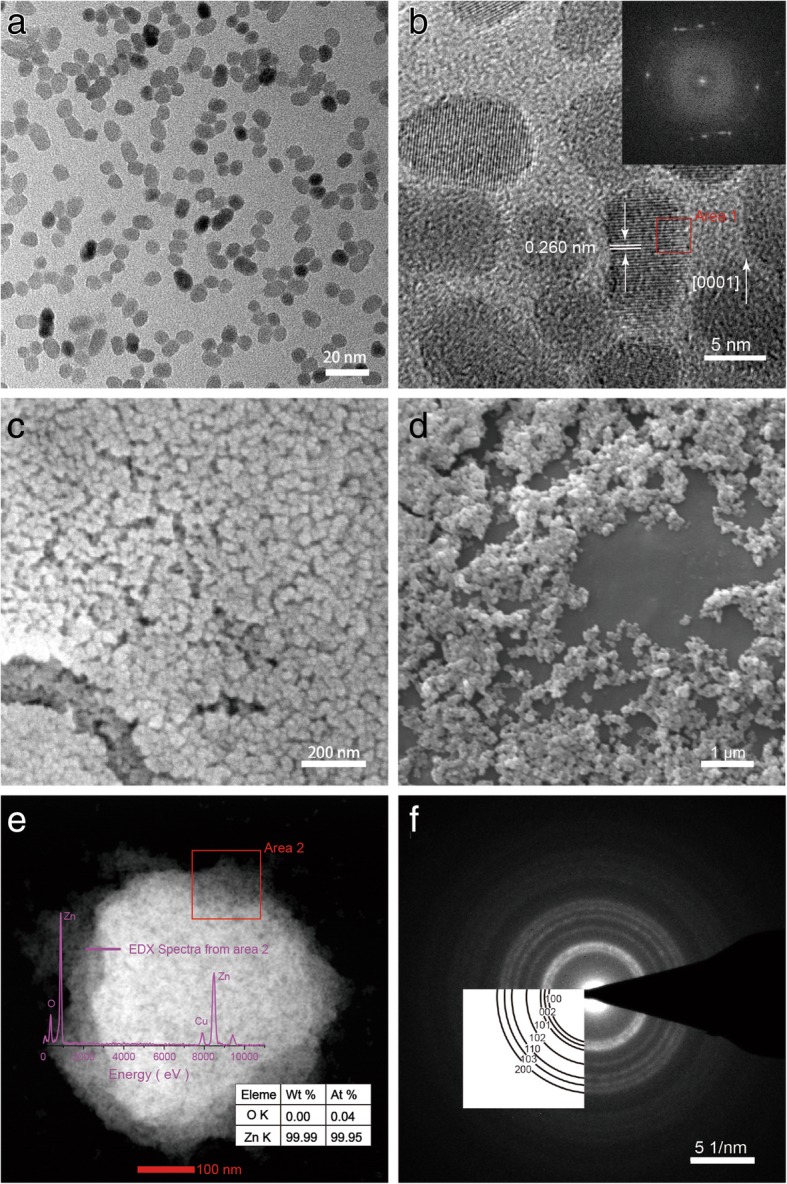
TEM, SEM, EDX, and SAED results for ZnO NPs from sample 4. **a**, **b** Low- and high-magnification TEM images showing bulk morphology of the sample and lattice fringes, respectively. **b** Fourier filtered image from area 1. **c**, **d** SEM images of well-dispersed particles. **e** SEM image of the particle used to record EDX spectrum from area 2. **f** SAED pattern shows wurtzite pattern

Electron microscopy is an excellent tool to characterize features associated with oriented attachment (OA) [12]. Morphology of ZnO NPs was spherical according to TEM results shown in Fig. 3. Fast Fourier transform (FFT) patterns (see inserts in Fig. 3b) clearly demonstrates single-crystal hexagonal structure with 2.60 Å spacing between two adjacent lattice fringes, which correspond to (002) planes of wurtzite [24]. SEM micrographs of particles from sample 4 show crystallite sizes larger than those determined by XRD and TEM probably because particles aggregated during sample preparation for SEM (see Fig. 3c, d). EDX spectra showed the presence of Zn (from ZnO NPs) and Cu (from the Cu grid used for sample preparation). SAED patterns are shown in Fig. 3b demonstrate crystalline nature of the samples.

**Fig. 4:**
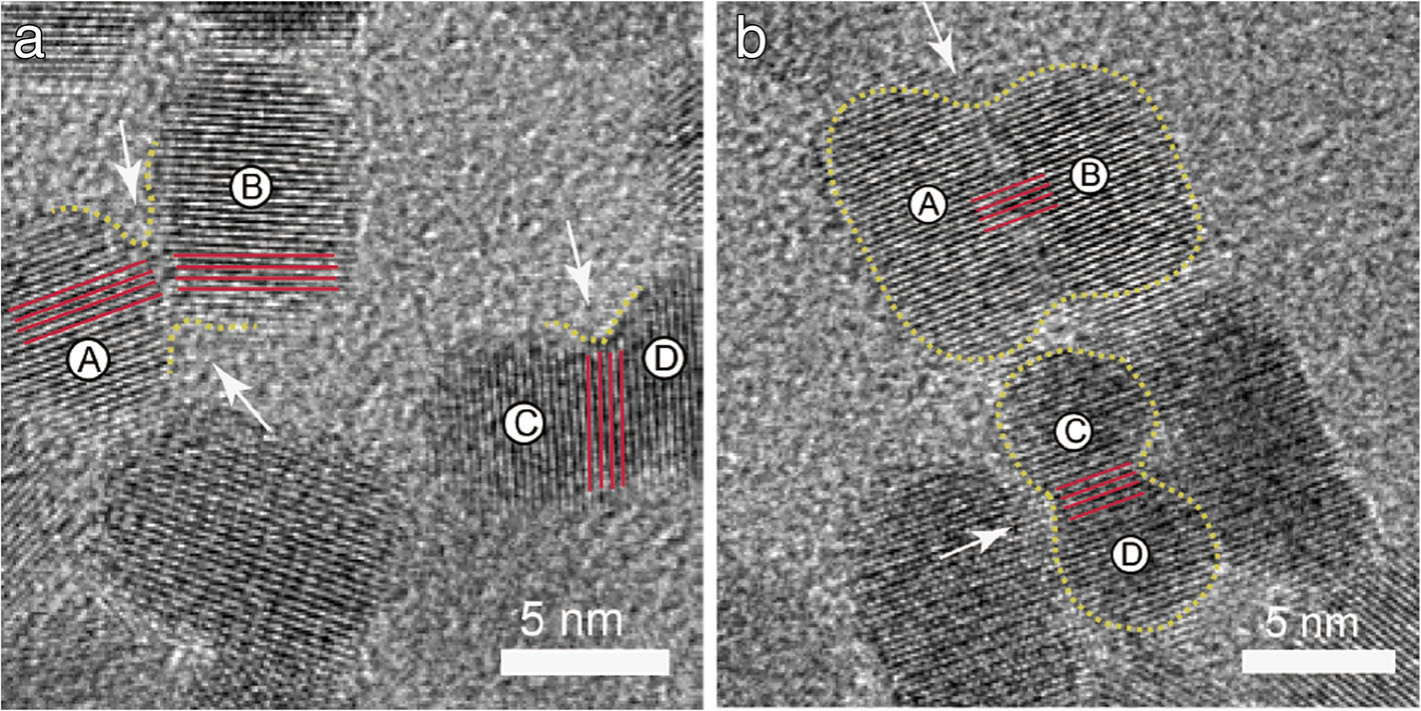
HRTEM images of ZnO NPs. **a** Dislocations resulted from the oriented attachment (OA) process. **b** Nanoparticles formed layer-by-layer either parallel or perpendicular to the *c*-axis of ZnO

Lattice planes of the merged particles were almost perfectly aligned. However, the bottlenecks and poorly merged fragments between the aligned dimers are still visible (see Fig. 4a). Small misalignments during grain and particle formation can lead to defects. Yet, these defects can be eliminated through recrystallization and rearrangement of ZnO NPs (see Fig. 4b). HRTEM shown in Fig. 4 shows that large particles formed by merging of adjacent particles. Areas A–B and C–D “aligned” into each other maintaining their perfect relative crystallographic orientation (see Fig. 4b). Dislocations formed among areas A–B and C–D (see Fig. 4a). Misorientation angles between blocks reported in previous literature were about several degrees [23].

TEM analysis revealed that nanorods formed by nanoparticles stacked layer-by-layer either parallel or perpendicular to the *c*-axis of wurtzite (see Fig. 4b). Adjacent nanoparticles were arranged either parallel or perpendicular like a wall [24]. No crystal defects were clearly observed as reaction time was prolonged, which also led to particles elongation along the *c*-axis of ZnO [25].

Process of oriented attachment (OA) depends on type of alcohol, water content in the solution and pressure [13, 26]. OA reaction is better controlled in ethanol in comparison to methanol. However, the most significant influence on OA process causes reaction time, temperature, and pH [27]. Our experiments showed that at 40.0 °C (and all other conditions equal to those for sample 21) no precipitation, confirming temperature importance for crystal growth. By varying these conditions, various shapes of particles can be obtained [28].

**Fig. 5:**
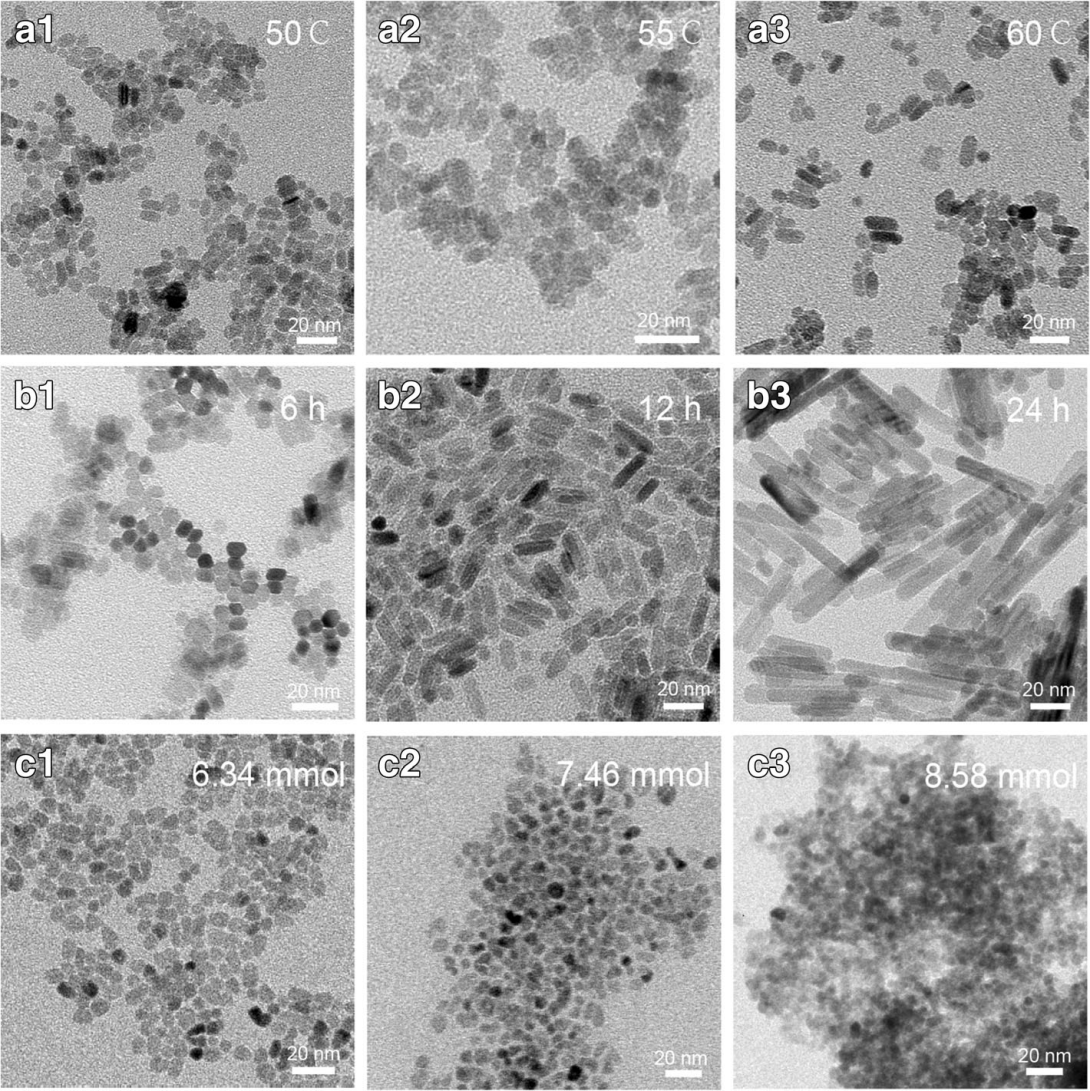
TEM images of ZnO NPs with different morphologies obtained by changing reaction **a** time and **b** temperature as well as **c** NaOH content. a1–3, b1–3, and c1–3 correspond to samples 2–4, sample 10–12, and sample 15–17, respectively. See Fig. 1 for a reference of these conditions

Results shown in Fig. 5 additionally confirm that growth of nanoparticle into nanorods was time- and temperature-dependent. Growth of nanoparticles was faster at higher temperatures. Change of reaction temperature resulted in some particle growth and blending. Most obvious temperature effects were observed for samples 2 and 4. When the reaction time was prolonged to 6 h, particles started to merge (see Fig. 5b). Further prolonging the reaction time to 12 h resulted in nanorods ~ 100 nm long and ~ 15 nm wide (see Fig. 5b). Strong (002) ZnO diffraction peak is consistent with ZnO NP shape observed by TEM. Both methods confirmed that the preferential growth direction for ZnO NP-oriented arrays was along the *c*-axis (see Fig. 5b).

**Fig. 6:**
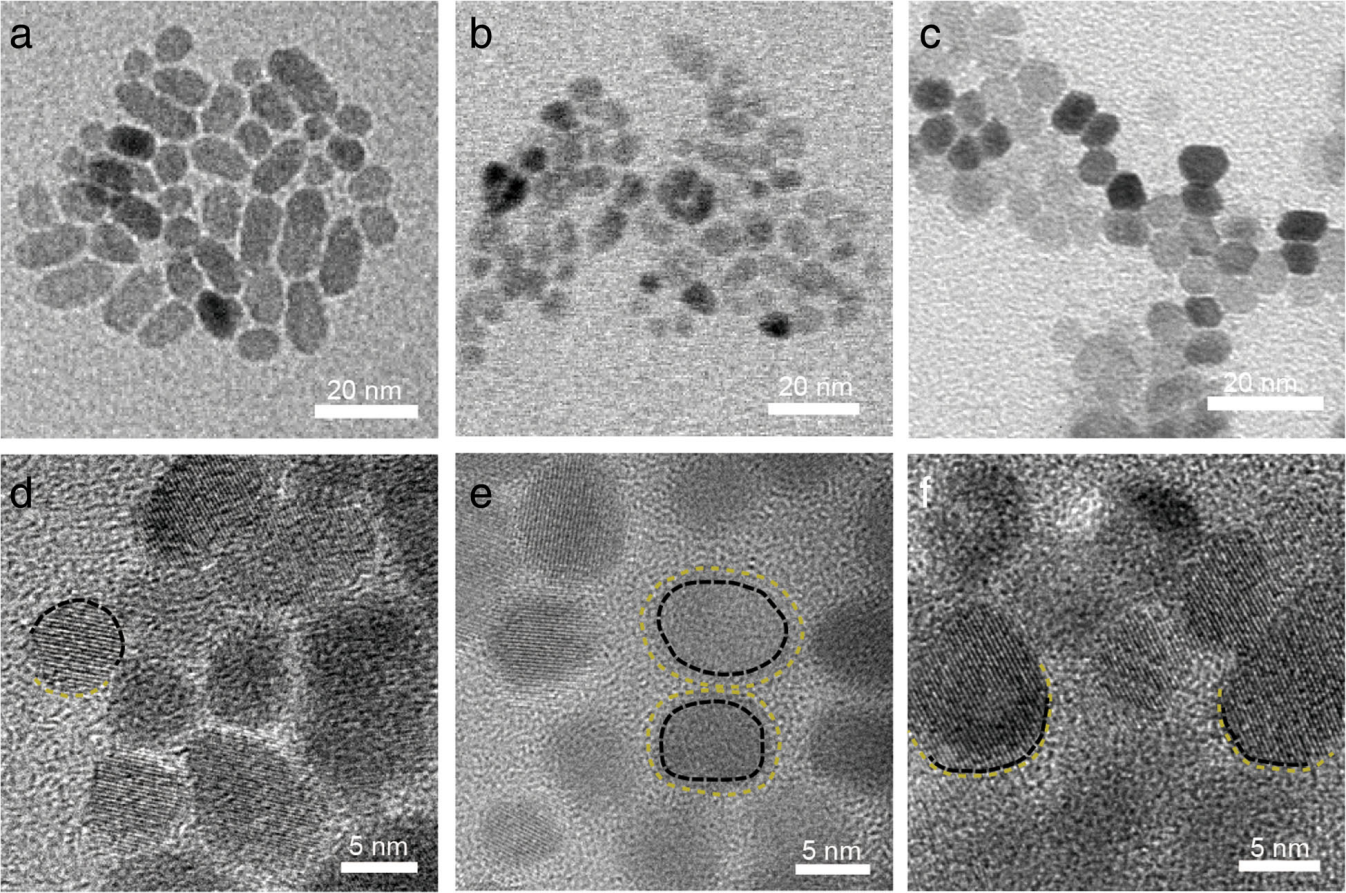
TEM images of ZnO NPs at different stages of the growth process. **a**, **d** Primary nucleation. **b**, **e** flake-like aggregates. **c**, **f** Well-crystallized particles

TEM demonstrated ZnO NPs with different stages of merging: starting with separation (see Fig. 6a) followed by mutual contact (see Fig. 6b) and complete merge (see Fig. 6c). These results provide data as well as evidence for OA mechanism analysis [29]. Changes of particles morphology can be observed as shown in Fig. 6d: typical features were the widening of the merging zone. When comparing to the images in Fig. 6f, adjacent nanoparticles in Fig. 6d demonstrated less visible merge line and a bigger merging zone. This is a direct evidence of changes occurring in particles prior to OA. These results clearly demonstrate that ZnO NPs underwent merging processes. “Rough” state (see Fig. 6c, d) on the surface of nanoparticles was observed for samples 3, 11, and 16 (see Fig. 1).

Although pH has little effect on the morphology of nanoparticles, the surface structure of the particles in these experiments changed (see Fig. 5c). The trend of morphological evolution indicated that rough states of particle surfaces are very likely the preliminary stages of the OA process; these particles represent intermediate species of the crystal growth [30, 31].

### DLS Analysis

**Fig. 7:**
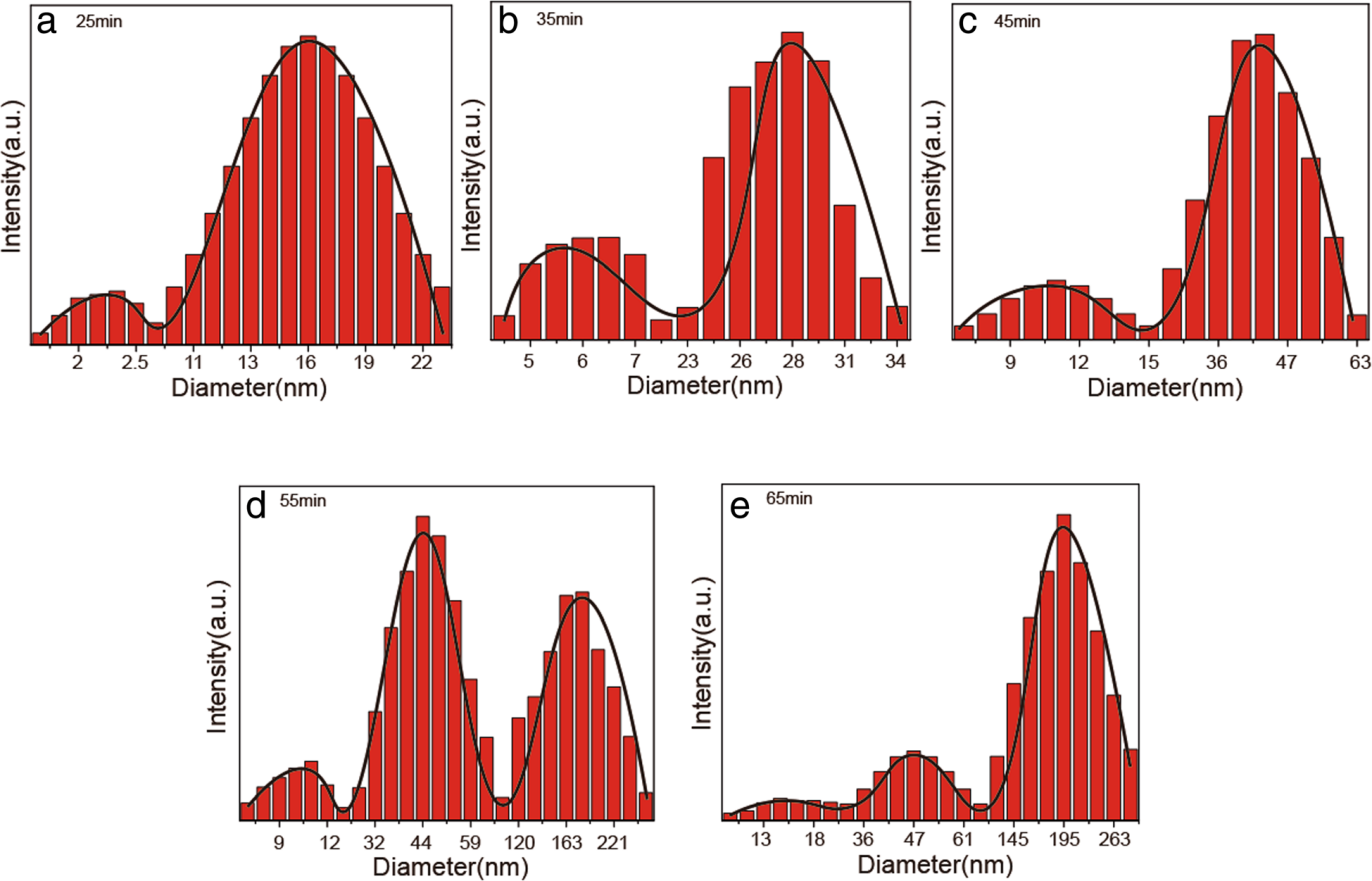
Particle size distribution of samples obtained at 55 °C, with 7.46 mmol and 0.1 mol of NaOH and zinc acetate dihydrate initial concentrations, respectively, and for **a** 25 min, **b** 35 min, **c** 45 min, **d** 55 min, and **e** 65 min

Figure 7 shows DLS data for ZnO NPs obtained at 55 °C, 7.46 mmol of NaOH, and 0.1 mmol of zinc acetate dihydrate at different reaction times. Polydispersity indices (PDI) for these samples ranged from 0.140 to 0.287. These changes reflect the evolution of ZnO NPs during the synthesis with different duration times. Figure 7a probably reflects particle states during the nucleation stage since Fig. 7b–e, which reflect changes during the later stages, demonstrate two particles, which are two times larger. This phenomenon hydrodynamically proves direct agglomeration of particles. It also illustrates the rationality of the bimodal size distribution, which is another evidence of the OA process [32, 33].

The bimodal distribution shown in Fig. 7 is also observed in the preparation process of other samples, i.e., sample 21 and 23, (Additional file 3: Figure S3). Typically, oriented attachment of nanoparticles is accompanied by a bimodal distribution of nanoparticle sizes [34]. This assumption was confirmed experimentally (based on TEM data) as a characteristic feature of OA [35].

Crystal growth is traditionally considered to be a spontaneous process. During nucleation by amorphism and polymorphism, the nuclei are thermodynamically stabilized by their strong tendency to minimize their surface energy [36]. Generally, the system preference is the growth of a single large particle. Obtained particle sizes are larger than those shown in the TEM images due to the aggregation of the particles [37].

However, bi- or polymodal distribution alone does not justify OA because similar results can be produced by usual aggregation of particles in a solution [38]. The most reliable and comprehensive approach to describe crystal growth mechanism is to analyze and to compare the results of several instrumental characterization methods.

### Analysis of Oriented Attachment

Classical crystal models (Ostwald ripening) states that nanoparticles originate through the formation of small crystalline nuclei in a supersaturated reaction solution followed by particles growth. Large nanoparticles will grow at the cost of the small ones to some extent. This mechanism is generally believed to be the main path of crystal growth in synthetic reaction systems [39]. Despite successes of classical crystallization theory [40], there are several phenomena associated with crystal growth that it cannot explain. One example is nucleation at low concentrations or unusual crystal suspension morphologies observed for synthetic ZnO NPs. These phenomena were attributed to non-classical crystal and growth models.

Typically, oriented attachment (OA), one of the non-classical theories of crystal growth, proceeds by repeating attachment events of merging particles on lattice-matched crystal facets [40, 41]. Many studies tried to identify the complete process of OA, as well as growth kinetics of nanoparticles and their assembles as well as merging processes. However, neither a comprehensive theory nor a definite conclusion has been proposed so far.

In this section, we examine emerging evidence for non-classical crystal growth of NPs and discuss the corresponding processes and mechanisms of nanoparticle formed by OA. Classical models describe clear boundaries dividing crystal particles from its monomeric building units. However, in the OA process, it becomes obvious that this boundary is not abrupt but rather represents a broad spectrum of intermediate structures from nanoparticles and to bulk crystals. Analysis of these “intermediate processes” converting small crystals to large crystals will help to understand changes that nano-surfaces undergo [42].

OA of particles resulting in the formation of larger aggregates and crystals is not a principally new concept [43]. Nevertheless, the mechanism of nanoparticle growth described during recent years often did not consider the OA process. Most studies neither considered structural changes of individual particles at these stages nor paid attention to OA processes on a macroscopic level [13].

In the solution with long-term suspension performance, if the particles are adjacent, the lattice planes will exhibit a more integrated trend (and not fusion). Thus, particle surface structure changes (similar to those shown in Fig. 6) seem to be the prerequisite to the crystallization step leading to particles crystallite fusion under experimental conditions similar to this work. According to the thermodynamic and dynamic mechanisms, the formation of stable phases in a solution should be preceded by the formation of metastable intermediate phases [44]. Recent studies demonstrated lower nucleation energy barrier (LNEB) than typically would be expected in the classical crystal model. LNEB might be attributed to the rough state of the particles [29].

The interplay between thermodynamics and kinetics leads to the main characteristics of oriented attachment (OA). Prior to the OA stage, nanoparticles grow and coarsen. When OA starts, the particle surface becomes smooth. When particles surfaces are atomically rough, the crystal growth rate is controlled by diffusion [29]. Such structural changes of nanoparticles might play an important role in promoting suspension properties, especially when particles are poorly dispersed. Understanding fusion structure is very important to study suspension properties of NPs because of their special structure and excellent stabilization in ethanol. Such NPs are almost similar to mesocrystals with enhanced and/or novel thermoelectric, photonic, catalytic, and photovoltaic properties [45, 46]. However, OA-grown NPs and mesocrystals are very different. This rough state does not contradict non-classical models of OA mechanisms but rather supplements them [47].

**Scheme 2:**
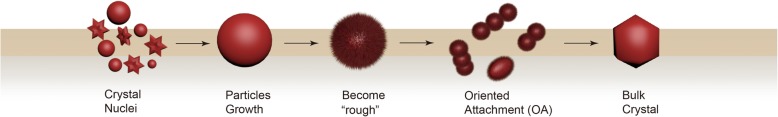
Stages of possible crystallization process-based OA

ZnO nanoparticle tends to cluster together, which is expected as the system tries to decrease its overall surface energy by matching crystal lattices and reducing exposed areas and defects. This typical process occurring during nanoparticle growth eventually changes particle surface structure [47]. Based on the main points of the discussion above, the mechanism for an oriented attachment (OA) during formation of suspension of ZnO NPs can be described as shown in Scheme 2.

Formation of bulk nanoparticles undergoes three major stages [36, 48]:

(i) Classical nucleation and crystal growth of the particles (nanocrystal formations);
(ii) Nanoparticles surface structure and morphology change (became the “rough” state);
(iii) Highly oriented aggregation between the nanoparticles (OA process).

According to this model, if the growth state of nanoparticles can be controlled at the rough stage, the overall suspension will maintain its dispersion for a long time. Developing crystal models for particle growth undergoing similar mechanisms will improve nanomaterial synthesis strategies. In addition, controlling the microstructure of synthetic materials using OA mechanisms is a promising and an insufficiently explored research area.

## Conclusions

This paper reports synthesis of ZnO NP suspension in ethanol and at low temperature without using any surfactants and/or dispersants. Such very stable suspensions were obtained by optimizing solution characteristics (temperature, aging time, precursor concentrations, and pH). Surface structures of ZnO NPs were mostly influenced by the reaction temperature, followed by reaction time and pH.

This work provides strong evidence that prior to oriented attachment (OA) process, the surface structure of adjacent particles transforms into a rough state, which changes material properties and its suspendability in the solution. It was shown for the first time that suspendability of ZnO NPs in ethanol can be controlled and further used in practical suspension-based applications.

This work opens a new way for understanding how structures of NPs influence their properties. Further and deeper understanding of OA also promises advances in various nanomaterial design and synthesis methods, which can be further used for diverse industrial applications.

## Additional files

Additional file 1: **Figure S1.** The absorbance versus wavelength curve of samples 3 and 21 (TIF 215 kb)

Additional file 2: **Figure S2.** XRD patterns of a sample 21 and b sample 23. XRD pattern of bulk ZnO (according to JCPDS no. 36-1451) is shown at the bottom of each set of XRD patterns (TIF 796 kb)

Additional file 3: **Figure S3.** Dynamic light scattering (DLS) measurements of a sample 21 and b sample 23 after 50 min (TIF 998 kb)

Additional file 4: **Figure S4.** Z-potentials of samples 3 and 21 (TIF 738 kb)

Additional file 5: **Table S1.** Sample identification (ID) as function of their preparation conditions. The reaction system for each sample had only a one-factor variable. Standard conditions were 60 °C, 2.25 h, 7.22 mmol of NaOH, and 3.73 mmol of zinc acetate dihydrate (DOC 50 kb)

## Data Availability

The datasets generated during and/or analyzed during the current study are available from the corresponding author on reasonable request.

## Abbreviations

DLS: Dynamic light scattering
EDX: Energy dispersive X-ray spectroscopy
HR-TEM: High-resolution transmission electron microscopy
LNEB: Lower nucleation energy barrier
NPs: Nanoparticles
OA: Oriented attachment
OR: Ostwald ripening
SAED: Selected area electron diffraction
SEM: Scanning electron microscope
TEM: Transmission electron microscopy
XRD: X-ray diffraction
