# Supplementary figures and images for: Preparation of ZnO Nanoparticles with High Dispersibility Based on Oriented Attachment (OA) Process
Source: Nanoscale Res Lett. 2019 Jun 20;14:210. doi: 10.1186/s11671-019-3038-3 (PMC6586737; doi:10.1186/s11671-019-3038-3)

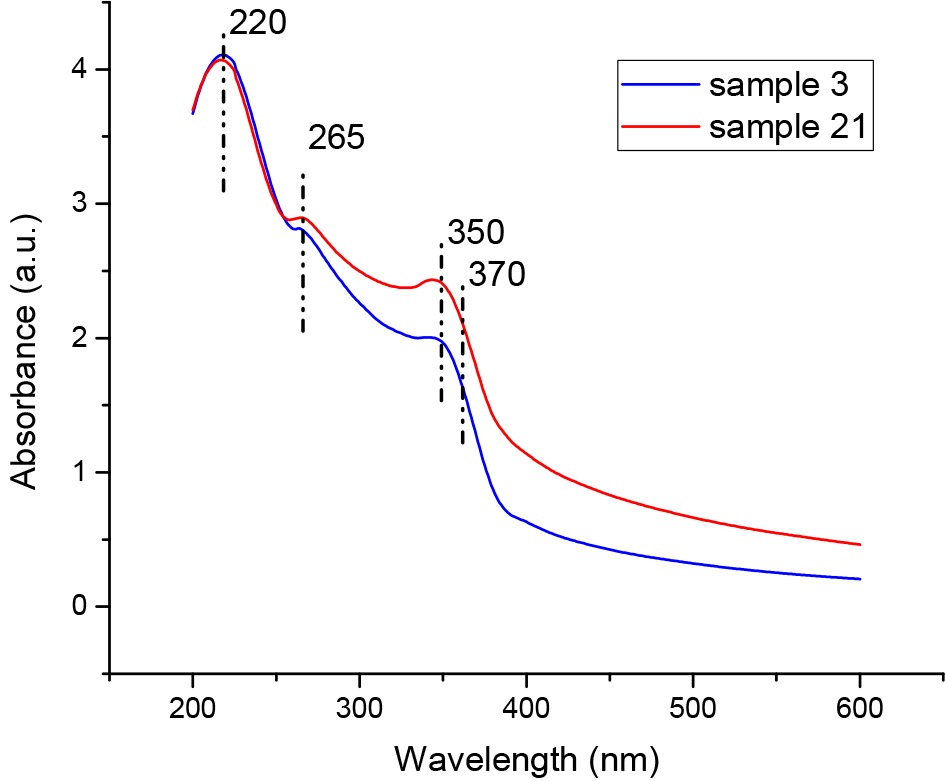

Supplement: Supplementary file 1 — Figure S1. The absorbance versus wavelength curve of samples 3 and 21 (TIF 215 kb) [file 11671_2019_3038_MOESM1_ESM.tif]

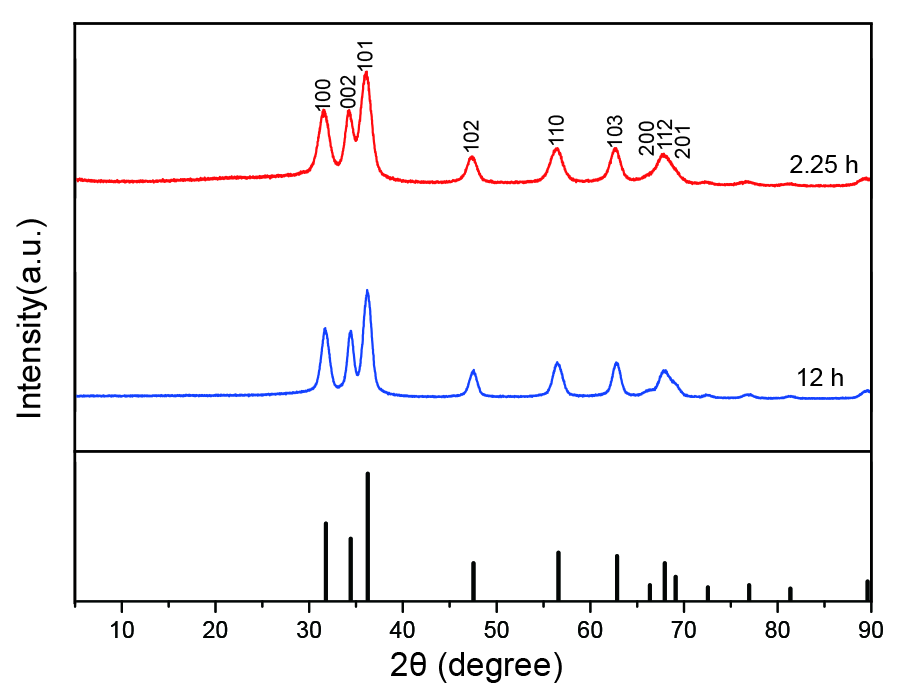

Supplement: Supplementary file 2 — Figure S2. XRD patterns of a sample 21 and b sample 23. XRD pattern of bulk ZnO (according to JCPDS no. 36-1451) is shown at the bottom of each set of XRD patterns (TIF 796 kb) [file 11671_2019_3038_MOESM2_ESM.tif]

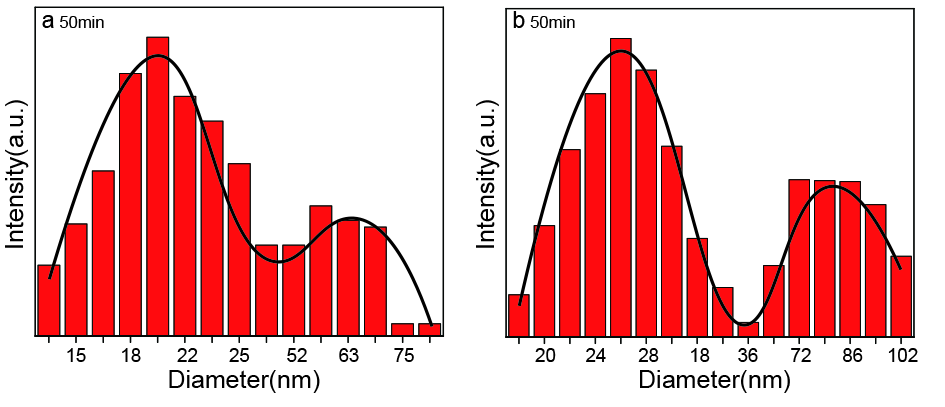

Supplement: Supplementary file 3 — Figure S3. Dynamic light scattering (DLS) measurements of a sample 21 and b sample 23 after 50 min (TIF 998 kb) [file 11671_2019_3038_MOESM3_ESM.tif]

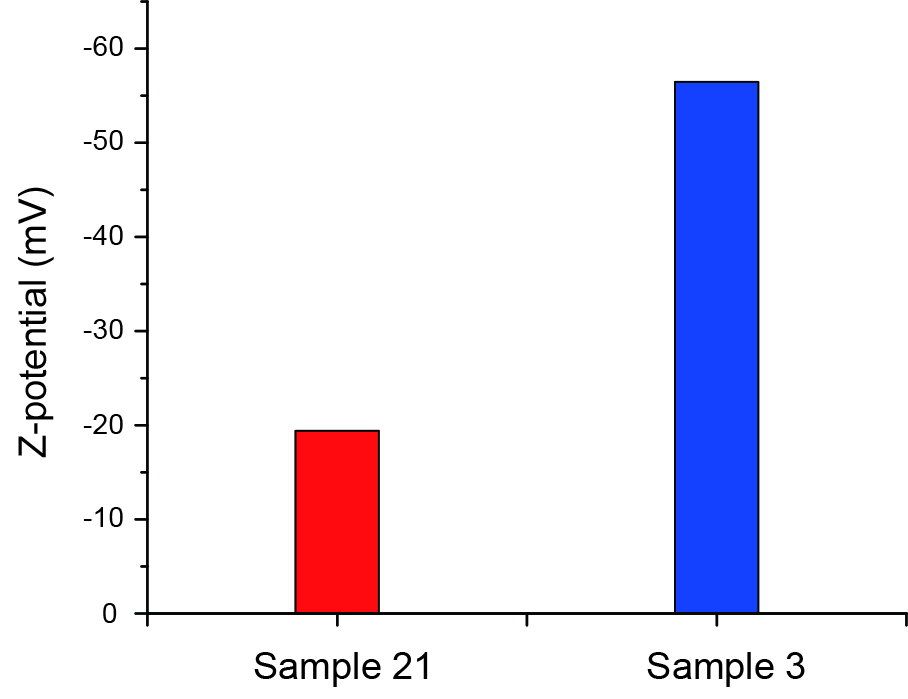

Supplement: Supplementary file 4 — Figure S4. Z-potentials of samples 3 and 21 (TIF 738 kb) [file 11671_2019_3038_MOESM4_ESM.tif]
