# Supplementary material for: Preparation of ZnO Nanoparticles with High Dispersibility Based on Oriented Attachment (OA) Process
Source: Nanoscale Res Lett. 2019 Jun 20;14:210. doi: 10.1186/s11671-019-3038-3 (PMC6586737; doi:10.1186/s11671-019-3038-3)
Supplement: Supplementary file 5 — Table S1. Sample identification (ID) as function of their preparation conditions. The reaction system for each sample had only a one-factor variable. Standard conditions were 60 °C, 2.25 h, 7.22 mmol of NaOH, and 3.73 mmol of zinc acetate dihydrate (DOC 50 kb) [file 11671_2019_3038_MOESM5_ESM.doc]

**Additional file:**

**Table S1.** Sample Identification (ID) as function of their preparation conditions. The reaction system for each sample had only one-factor variable. Standard conditions were 60℃, 2.25 h, 7.22 mmol of NaOH and 3.73 mmol of zinc acetate dihydrate.

| **Identification (IDs)**  **Variable** | **Temperature(℃)** | **Zinc acetate dihydrate(mmol)** | **NaOH(mmol)** | **Time(h)** |
| --- | --- | --- | --- | --- |
| **Samples 1** | 45℃ | 3.73 | 7.22 | 2.25 h |
| **Samples 2** | 50℃ | 3.73 | 7.22 | 2.25 h |
| **Samples 3** | 55℃ | 3.73 | 7.22 | 2.25 h |
| **Samples 4** | 60℃ | 3.73 | 7.22 | 2.25 h |
| **Samples 5** | 65℃ | 3.73 | 7.22 | 2.25 h |
| **Samples 6** | 70℃ | 3.73 | 7.22 | 2.25 h |
| **Samples 7** | 60℃ | 1 | 7.22 | 2.25 h |
| **Samples 8** | 60℃ | 4 | 7.22 | 2.25 h |
| **Samples 9** | 60℃ | 7 | 7.22 | 2.25 h |
| **Samples 10** | 60℃ | 10 | 7.22 | 2.25 h |
| **Samples 11** | 60℃ | 14 | 7.22 | 2.25 h |
| **Samples 12** | 60℃ | 18 | 7.22 | 2.25 h |
| **Samples 13** | 60℃ | 3.73 | 3.73 | 2.25 h |
| **Samples 14** | 60℃ | 3.73 | 5.22 | 2.25 h |
| **Samples 15** | 60℃ | 3.73 | 6.34 | 2.25 h |
| **Samples 16** | 60℃ | 3.73 | 7.46 | 2.25 h |
| **Samples 17** | 60℃ | 3.73 | 8.58 | 2.25 h |
| **Samples 18** | 60℃ | 3.73 | 9.33 | 2.25 h |
| **Samples 19** | 60℃ | 3.73 | 7.22 | 1h |
| **Samples 20** | 60℃ | 3.73 | 7.22 | 1.5h |
| **Samples 21** | 60℃ | 3.73 | 7.22 | 2.25h |
| **Samples 22** | 60℃ | 3.73 | 7.22 | 6h |
| **Samples 23** | 60℃ | 3.73 | 7.22 | 12h |
| **Samples 24** | 60℃ | 3.73 | 7.22 | 24h |
